# Non-Disruptive Tactics of Suppression Are Superior in Countering Terrorism, Insurgency, and Financial Panics

**DOI:** 10.1371/journal.pone.0018545

**Published:** 2011-04-13

**Authors:** David A. Siegel

**Affiliations:** Department of Political Science, Florida State University, Tallahassee, Florida, United States of America; University of Swansea, United Kingdom

## Abstract

**Background:**

Suppressing damaging aggregate behaviors such as insurgency, terrorism, and financial panics are important tasks of the state. Each outcome of these aggregate behaviors is an emergent property of a system in which each individual's action depends on a subset of others' actions, given by each individual's network of interactions. Yet there are few explicit comparisons of strategies for suppression, and none that fully incorporate the interdependence of individual behavior.

**Methods and Findings:**

Here I show that suppression tactics that do not require the removal of individuals from networks of interactions are nearly always more effective than those that do. I find using simulation analysis of a general model of interdependent behavior that the degree to which such less disruptive suppression tactics are superior to more disruptive ones increases in the propensity of individuals to engage in the behavior in question.

**Conclusions:**

Thus, hearts-and-minds approaches are generally more effective than force in counterterrorism and counterinsurgency, and partial insurance is usually a better tactic than gag rules in quelling financial panics. Differences between suppression tactics are greater when individual incentives to support terrorist or insurgent groups, or susceptibilities to financial panic, are higher. These conclusions have utility for policy-makers seeking to end bloody conflicts and prevent financial panics. As the model also applies to mass protest, its conclusions provide insight as well into the likely effects of different suppression strategies undertaken by authoritarian regimes seeking to hold on to power in the face of mass movements seeking to end them.

## Introduction

States or other actors desiring to suppress damaging aggregate behaviors including insurgency, terrorism, and financial panics have a vital interest in the relative efficacy of different tactics of suppression. Further, these same actors may seek to understand the spread and likelihood of success of mass movements aimed at toppling authoritarian regimes. However, comparisons of tactics are rare [1]–[6], and none fully account for the manner in which the spread of these behaviors depends on both individual susceptibility to the behavior and the degree to which individual behavior is interdependent [7]–[13]. I analyze a general model of the suppression of interdependent behavior that compares the two primary mechanisms of suppression: altering individual susceptibility, which I call non-disruptive; and reducing exposure to those already taking part in the behavior, which I call disruptive.

## Methods

Individuals in the model are described by three variables. The first represents individual susceptibility to support terrorism or insurgency, participate in protests or mass movements, or succumb to financial panic. This variable may be a function, for example, of interests, private information, or economic status. Examples of populations with high susceptibility towards participation in aggregate behaviors designed to alter actions by the state might include African-Americans in parts of the American South during the late 1950s or working class Catholics in Belfast in 1969. The second variable represents exposure to financial panic, or to the ideas, information, and influence of those supporting terrorism or insurgency or participating in protests or mass movements. The third specifies whether one supports a terrorist or insurgent group, participates in a protest or mass movement, or panics during a financial crisis and attempts to withdraw money from the system. I label *participation* any of support, participation, or panic, and call the proportion of the population participating the participation rate. The model (detailed in Supporting Information File S1 section 1) assumes that exposure is increasing in the participation rate [7], [9], [13] at a rate proportional to the transmissibility of the behavior. If exposure exceeds a threshold determined by susceptibility, an individual participates. Results below assume that individuals can cease participation of their own accord should exposure again drop below this threshold. This is the natural assumption for support for terrorism or insurgency, participation in protests or mass movements, and participation in some financial panics, but nothing substantive changes if participation cannot be revoked (see Supporting Figs. S4, S5, and S6), the natural assumption for most financial panics. The population size in all results below is 1,000 individuals (see Supporting Fig. S2 for the effect of varying population size).

I consider two suppression tactics mirroring the two mechanisms for suppression. The first I call disruptive, as it removes individuals from the population to eliminate the effect of their participation on others. This approach typically involves military or police force in insurgency, terrorism, protests, or mass movements, and entails collateral psychological or economic damage [14]–[17]. Force eliminates supporters and reduces exposure to supporters' ideas, influence, and information. Examples include imprisonment, deportation, rendition, and assassination. Stops on trading coupled with gag rules [18] serve as disruptive suppression in financial panics, as they prevent the further influence of the participant. Disruptive suppression is implemented in the model by the removal of participating individuals at a rate corresponding to the strength of suppression.

The second tactic of suppression I denote non-disruptive as it alters susceptibility rather than removes individuals. In counterinsurgency or counterterrorism this is often referred to as a hearts-and-minds approach; the same term could easily be applied in countering protests or mass movements. Disincentives to individual support include institutional and infrastructure development, job creation, and education. The primary mode of suppression of bank runs and other financial panics [19], [20] is insurance. Partial insurance or government bailouts are non-disruptive tactics that alter susceptibility to panic by reducing the cost of financial collapse. Non-disruptive suppression is implemented in the model by altering all individuals' susceptibilities at a rate corresponding to the strength of suppression.

**Figure 1:**
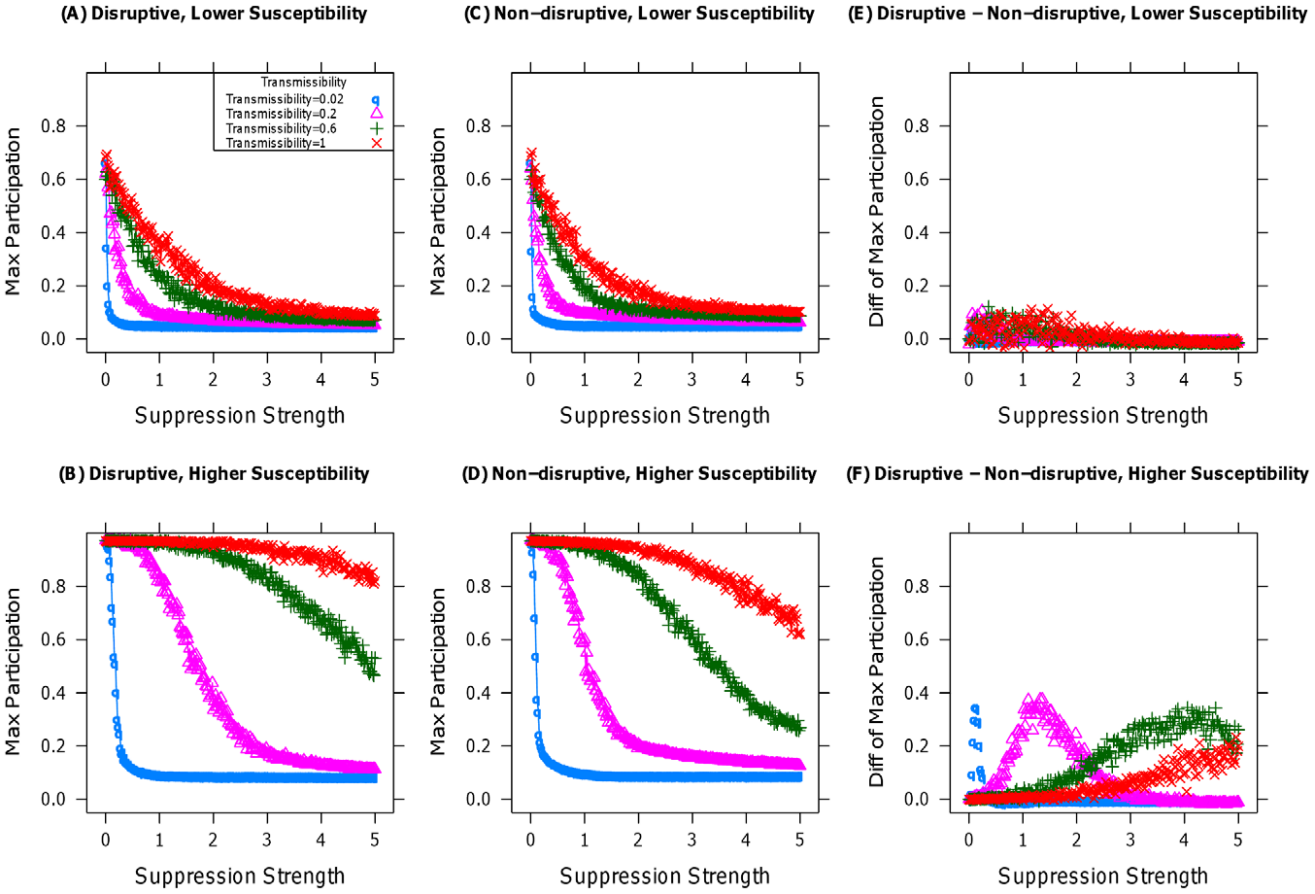
Effect of suppression on maximum participation level. (A) and (B) display the effect of disruptive tactics on maximum participation levels. (C) and (D) display the effect of non-disruptive tactics on the same. (A) and (C) use populations with lower susceptibilities than (B) and (D); each curve represents a different level of transmissibility. (E) and (F) display the differences between participation under disruptive and non-disruptive tactics (subtracting (C) from (A) and (D) from (B)), using respectively populations with lower and higher susceptibilities.

The order of operations in the model (see Supporting Fig. S1) is: 1) Susceptibilities are distributed across the population, 2) The level of exposure updates based on the participation rate, 3) Individuals make participation decisions based on a comparison between their susceptibilities and exposure, 4) Suppression occurs, and 5) Steps 2–4 repeat until participation is zero. I analyze the model using simulation (see Supporting Information File S1 section 2) and find consistent evidence that non-disruptive tactics are generally more efficacious than disruptive ones. Fig. 1 displays the mean response of the maximum participation rate achieved during a simulation history to the strength of suppression. I examine the maximum as it captures the point of greatest threat. Different curves in a plot correspond to different levels of transmissibility. The scales for suppressive strength were chosen so that a reduction of susceptibility produces roughly the same instantaneous change to one's behavior as does another's removal, at full population with maximal transmissibility. Plots labeled lower susceptibility make use of a population with fewer very susceptible individuals than those labeled higher susceptibility (see Supporting Information File S1 section 2).

## Results

Figs. 1A–D display the effects of disruptive and non-disruptive suppression on maximal participation in populations with lower and higher susceptibility; Figs. 1E and 1F display the differences in participation under disruptive and non-disruptive suppression in each case. Though the functional dependence of participation on suppression strength is similar in both cases, Figs. 1E and 1F indicate that non-disruptive suppression is significantly more effective, with each level of transmissibility possessing a suppression strength at which the benefits of non-disruptive suppression are the most substantial. Only when both tactics are extremely effective can disruptive tactics compete. Non-disruptive tactics better take advantage of positive feedbacks in interdependent behavior, reducing susceptibility without increasing the relevance of remaining participants, as does the reduction in the population caused by disruptive tactics. This is particularly important under high susceptibility, when early participation is more widespread. These results hold even more strongly when considering the mean level of support for terrorism or insurgency, or participation in protests or mass movements, where coopting participants can be a powerful tool (see Supporting Fig. S3).

**Figure 2 pone-0018545-g002:**
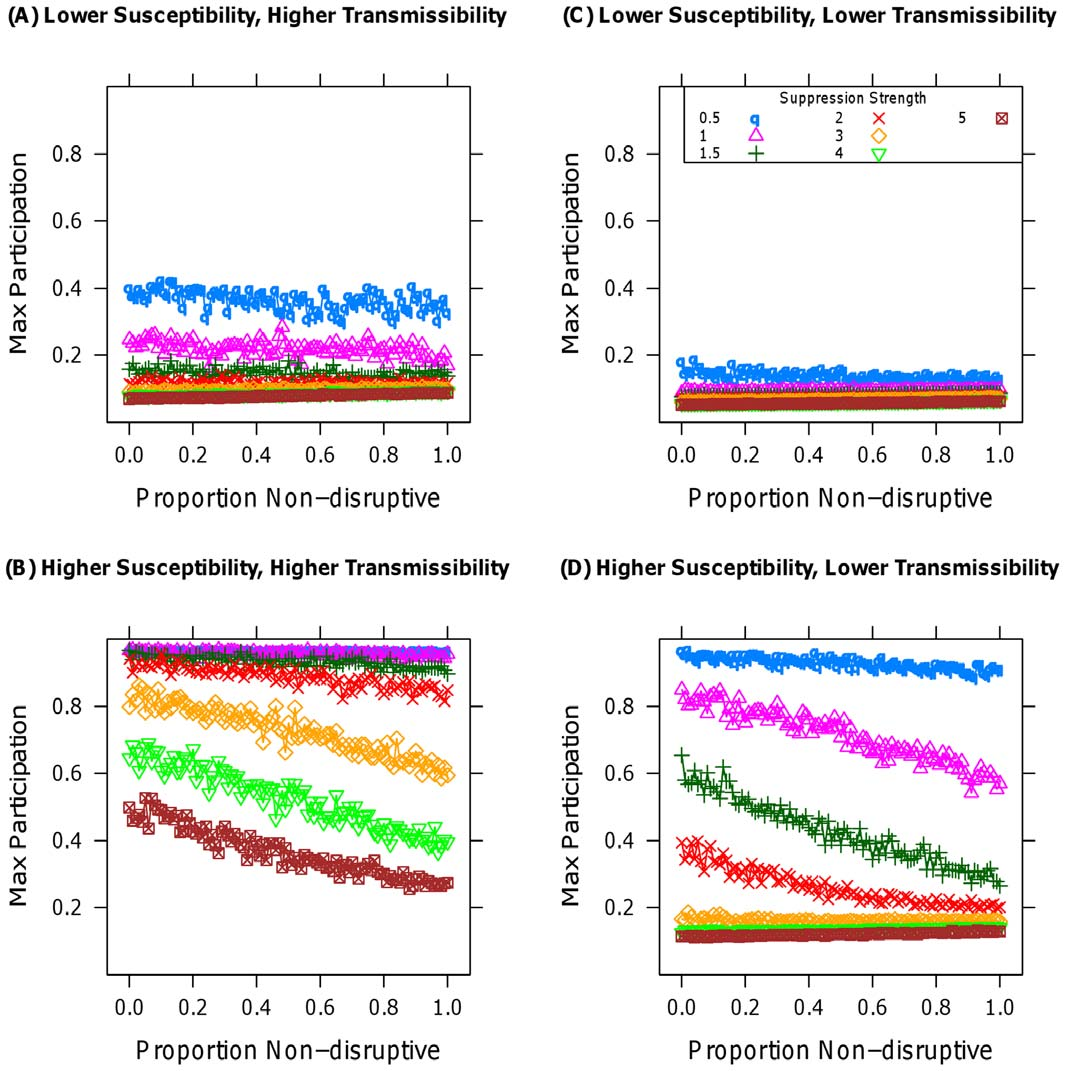
Aggregate maximal participation plotted against the proportion of the total suppression strength applied to non-disruptive tactics. Each curve represents a different total suppression strength. (A) and (C) use a population with lower susceptibilities than (B) and (D). (A) and (B) use greater transmissibility than (C) and (D).

Fig. 1 does not imply a lack of complementarity between the two tactics, so in Fig. 2 I apply both tactics simultaneously to see if a combination might be superior. To avoid conflating nonlinearities arising from increasing suppressive strength and from complementarities, I keep total suppressive strength fixed while varying the proportion given to non-disruptive tactics, for several levels of suppressive strength. Fig. 2 illustrates that increasing the proportion of non-disruptive tactics either has little effect on participation or strictly decreases it. Whenever non-disruptive tactics are better than disruptive ones individually, no combination of the two is superior to pure non-disruption. I note that this scenario is the best case for the use of disruptive tactics, as I am assuming they do not lead the population to distrust non-disruptive approaches.

**Figure 3 pone-0018545-g003:**
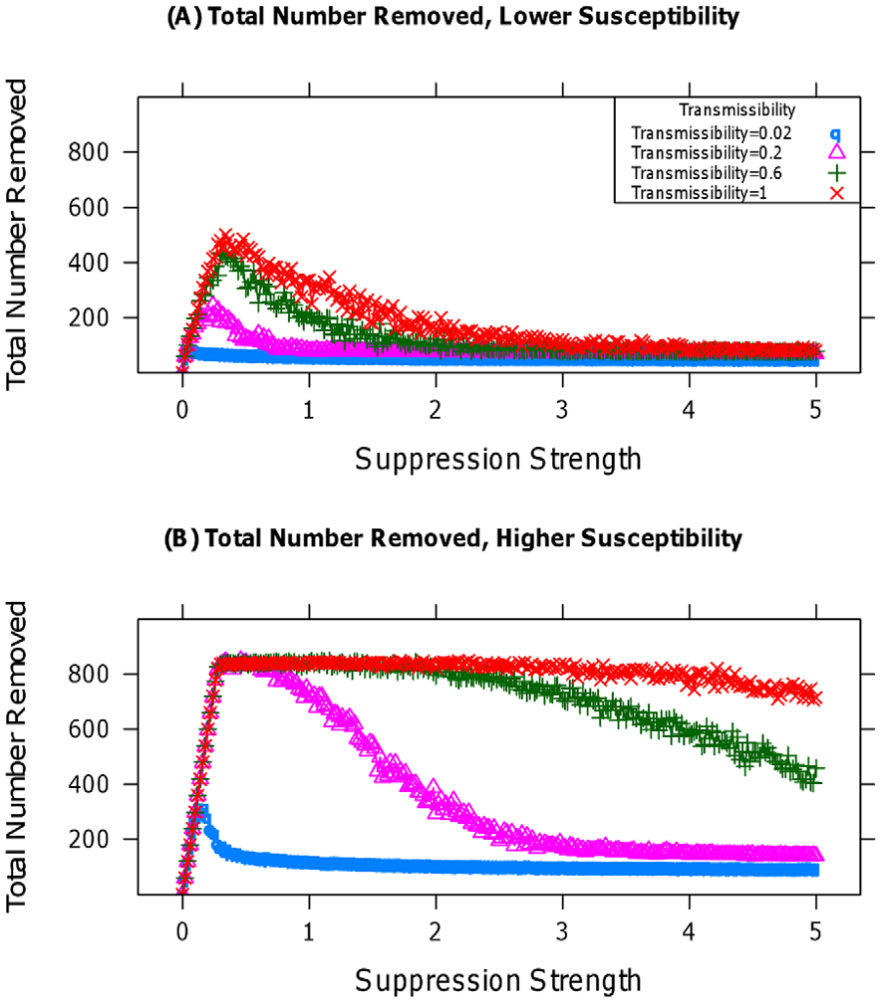
Number of individuals removed. (A) and (B) display the number of individuals removed during the period of nonzero participation. (A) uses a population with lower susceptibilities than (B). Each curve assumes a different level of transmissibility.

States desiring to suppress participation may care as much about cost-effectiveness as they do the functional response to suppression. The above analysis does not address this, as it does not specify the cost of one unit of suppression arising from either tactic. Thus, I perform two additional analyses. First, I note that disruptive tactics may entail additional logistical costs, increasing in the number of individuals removed. Figs. 3A and 3B display the total number of individuals removed from the population in the course of bringing the level of participation to zero, under lower and higher susceptibility respectively. As Fig. 3 indicates, the number removed can be significant, particularly when susceptibility is high. Further, in all cases the total number removed is non-monotonic in the rate of suppression; sufficiently fast removal prevents early participants from affecting others, reducing the overall number removed. In cases in which logistical, financial, or moral costs prevent sufficiently quick action, the total number of people eventually affected by state action greatly increases, potentially leading to substantial induced costs. Thus, non-disruptive tactics are likely to become relatively more cost-effective when rapid state action is impeded.

**Figure 4:**
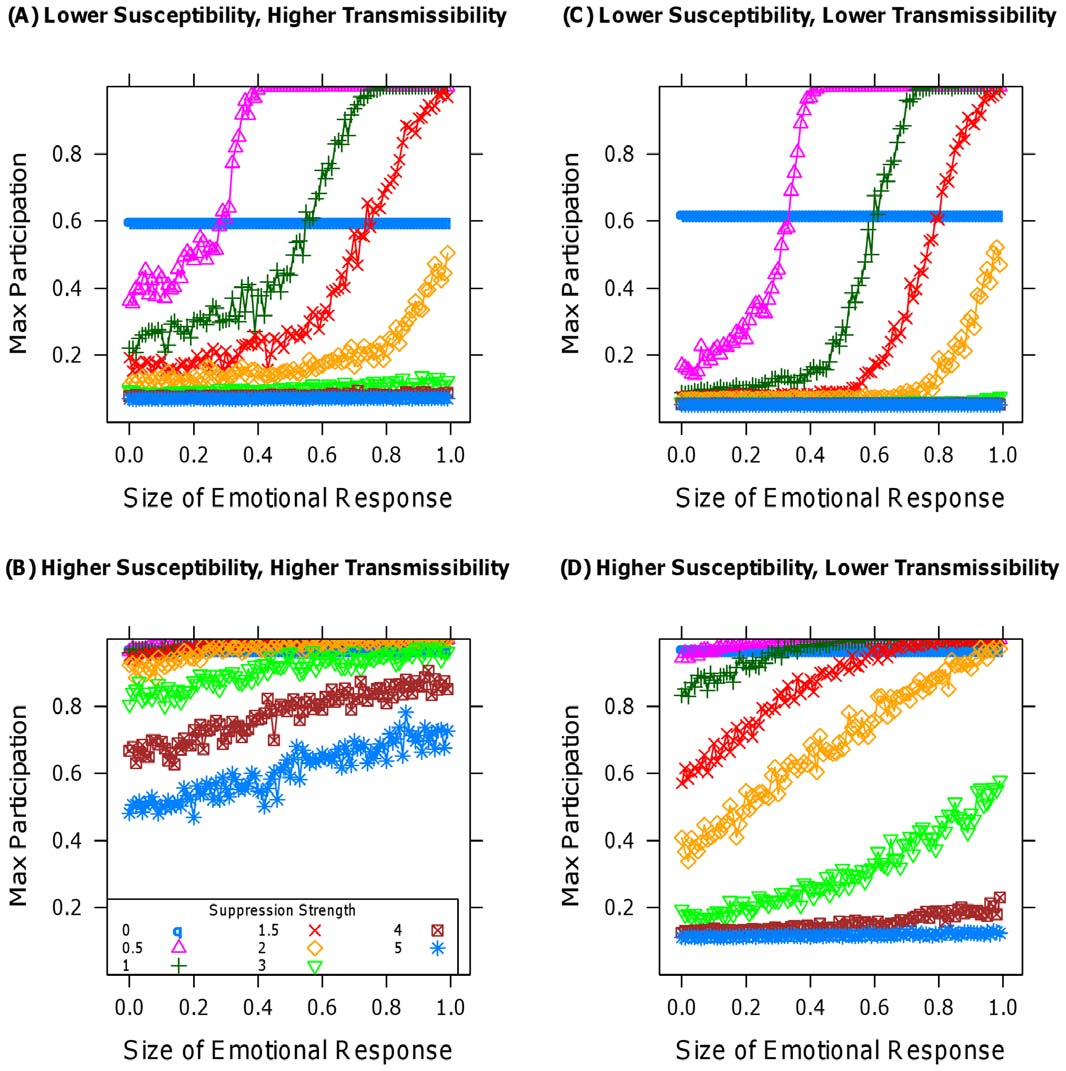
Aggregate maximal participation plotted against the size of the emotional response to the removal of others. Each curve represents a different removal rate. (A) and (C) use a population with lower susceptibilities than (B) and (D). (A) and (B) use greater transmissibility than (C) and (D). The horizontal line corresponding to no removal is a baseline.

Second, I note that disruptive tactics can lead to anger-induced micromobilization [21], [22], as people respond emotionally to the removal of others. Unlike the other results, this is primarily an issue for support for terrorism or insurgency, or participation in protests or mass movements, though one could imagine that gag rules and bans on selling might exacerbate panic in that they are signals of the weakness of the financial system. Fig. 4 explores the effect of varying the strength of emotional, angry responses to removal, modeled as an increase in susceptibility that occurs after each removal. The horizontal axes are one-fifth the scale of those in the plots in Fig. 1, each line represents a different level of suppression, and the horizontal line corresponding to no removal provides a baseline. Any level of participation greater than this line indicates a counterproductive action on the part of the state, leading to backlash by the population that is worse for the state than the original collective action would have been in the absence of suppression. Figs. 4B and 4D indicate that under higher susceptibility the addition of anger diminishes the efficacy of suppression but does not render it counterproductive. In contrast, Figs. 4A and 4C illustrate that under lower susceptibility, unless suppressive strength is very high, anger plays a strong, nonlinear role, and suppression becomes counterproductive in many cases.

## Discussion

Considered together, the results of Figs. 1–4 provide strong evidence of the superiority of non-disruptive tactics of suppression, particularly when susceptibilities are comparatively high or the possibility of an emotional response to disruptive tactics is present. Table 1 provides a concise summary of these results, along with the effect of variation in the model's parameters. The model's conclusions suggest expanded use of hearts and minds in counterterror and counterinsurgency operations at the expense of force, and the utility of both institutionalized insurance and government bailouts for limiting financial panics. They also imply that only the most brutal crackdowns by threatened authoritarian regimes would be more effective at maintaining the regime's power than would be a less disruptive, less violent approach. Finally, the generality and expandability of the simulation model, described fully in the Supporting Information File S1, provides a jumping off point for further exploration of the effect of suppression on collective action in other contexts [23] in a way that would be far more difficult in an analytic, closed-form setting, as seen in Section 2.1 of the Supporting Information File S1.

**Table 1:** Summary of Results.

Parameter Increase	Participation Rate without Suppression	Participation Rate with Suppression	Relative Benefit of Non-disruptive Suppression
Susceptibility	Increases	Increases	Increases
Transmissibility	Unchanged	Increases	Alters effect of increasing Suppression Strength on
Suppression Strength	N/A	Decreases	Increases, then Decreases
Size of Emotional, Angry Response	N/A	Increases	Increases

Columns 2–4 display the effect of increases in the parameter in the first column on, respectively: the participation rate when there is no suppression, the participation rate when there is suppression, and the degree to which non-disruptive suppression tactics are more effective than disruptive suppression tactics.

## Supporting Information

Figure S1 Schematic of the order of operations of the model
(TIFF)

Figure S2 Kernel density plots for the maximal participation level under two rates of removal: (A) Low (x = 0.0005 N) and (B) High (x = 0.005 N). (A) and (B) each contain six subplots that vary N and σ.
(TIFF)

Figure S3 Effect of suppression on mean participation level. (A) and (B) display the effect of disruptive tactics on mean participation levels. (C) and (D) display the effect of non-disruptive tactics on the same. (A) and (C) use populations with lower susceptibilities than (B) and (D); each curve represents a different level of transmissibility. (E) and (F) display the differences between participation under disruptive and non-disruptive tactics (subtracting (C) from (A) and (D) from (B)), using respectively populations with lower and higher susceptibilities.
(TIFF)

Figure S4 Effect of suppression on maximum participation level; no one can cease participating. (A) and (B) display the effect of disruptive tactics on maximum participation levels. (C) and (D) display the effect of non-disruptive tactics on the same. (A) and (C) use populations with lower susceptibilities than (B) and (D); each curve represents a different level of transmissibility. (E) and (F) display the differences between participation under disruptive and non-disruptive tactics (subtracting (C) from (A) and (D) from (B)), using respectively populations with lower and higher susceptibilities.
(TIFF)

Figure S5 Aggregate maximal participation plotted against the proportion of the total suppression strength applied to non-disruptive tactics; no one can cease participating. Each curve represents a different total suppression strength. (A) and (C) use a population with lower susceptibilities than (B) and (D). (A) and (B) use greater transmissibility than (C) and (D).
(TIFF)

Figure S6 Number of individuals removed; no one can cease participating. (A) and (B) display the number of individuals removed during the period of nonzero participation. (A) uses a population with lower susceptibilities than (B). Each curve assumes a different level of transmissibility.
(TIFF)

File S1 Supporting Information
(PDF)
