# Supplementary figures and images for: Non-Disruptive Tactics of Suppression Are Superior in Countering Terrorism, Insurgency, and Financial Panics
Source: PLoS One. 2011 Apr 13;6(4):e18545. doi: 10.1371/journal.pone.0018545 (PMC3076430; doi:10.1371/journal.pone.0018545)

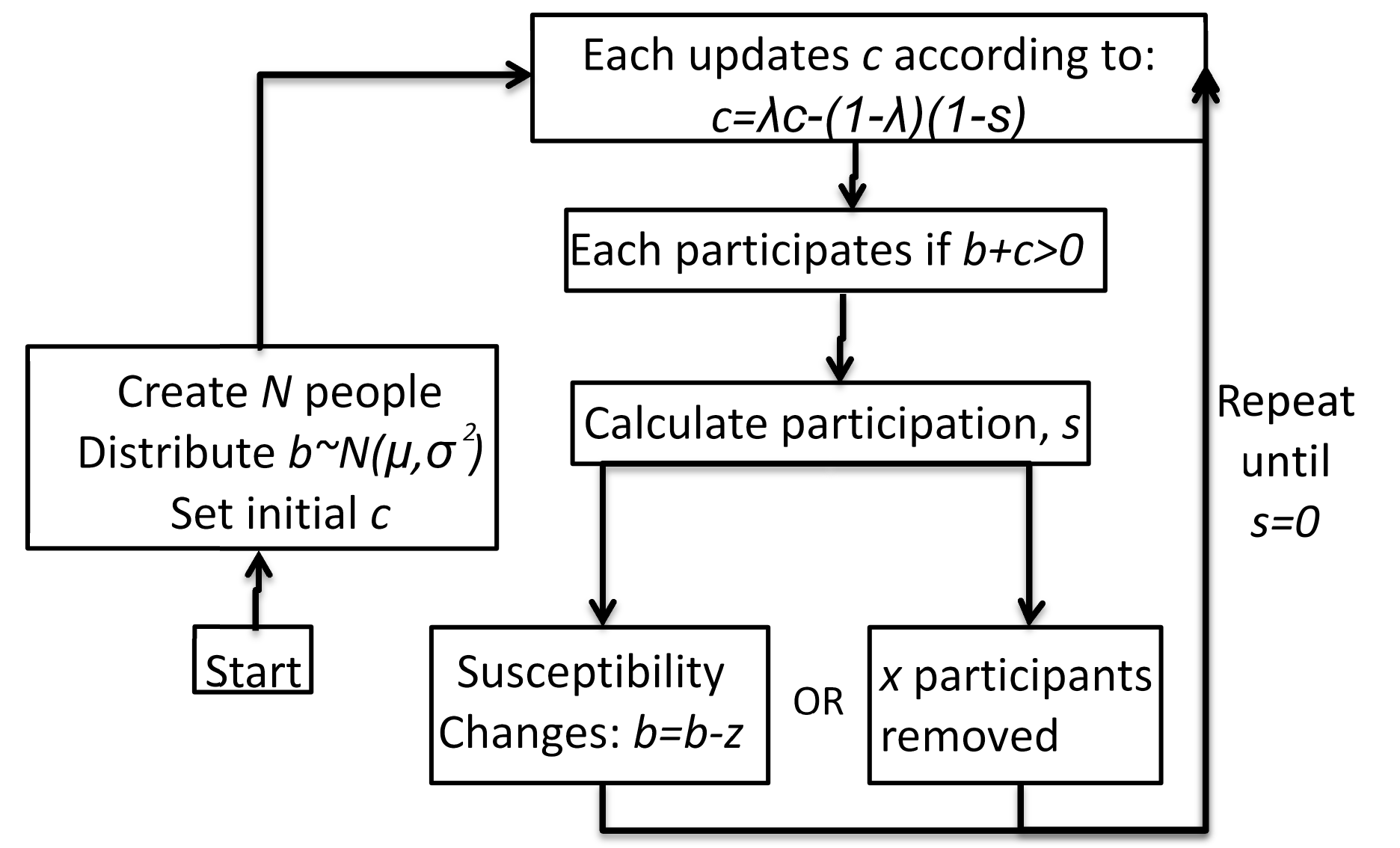

Supplement: Figure S1 — Schematic of the order of operations of the model (TIFF) [file pone.0018545.s001.tif]

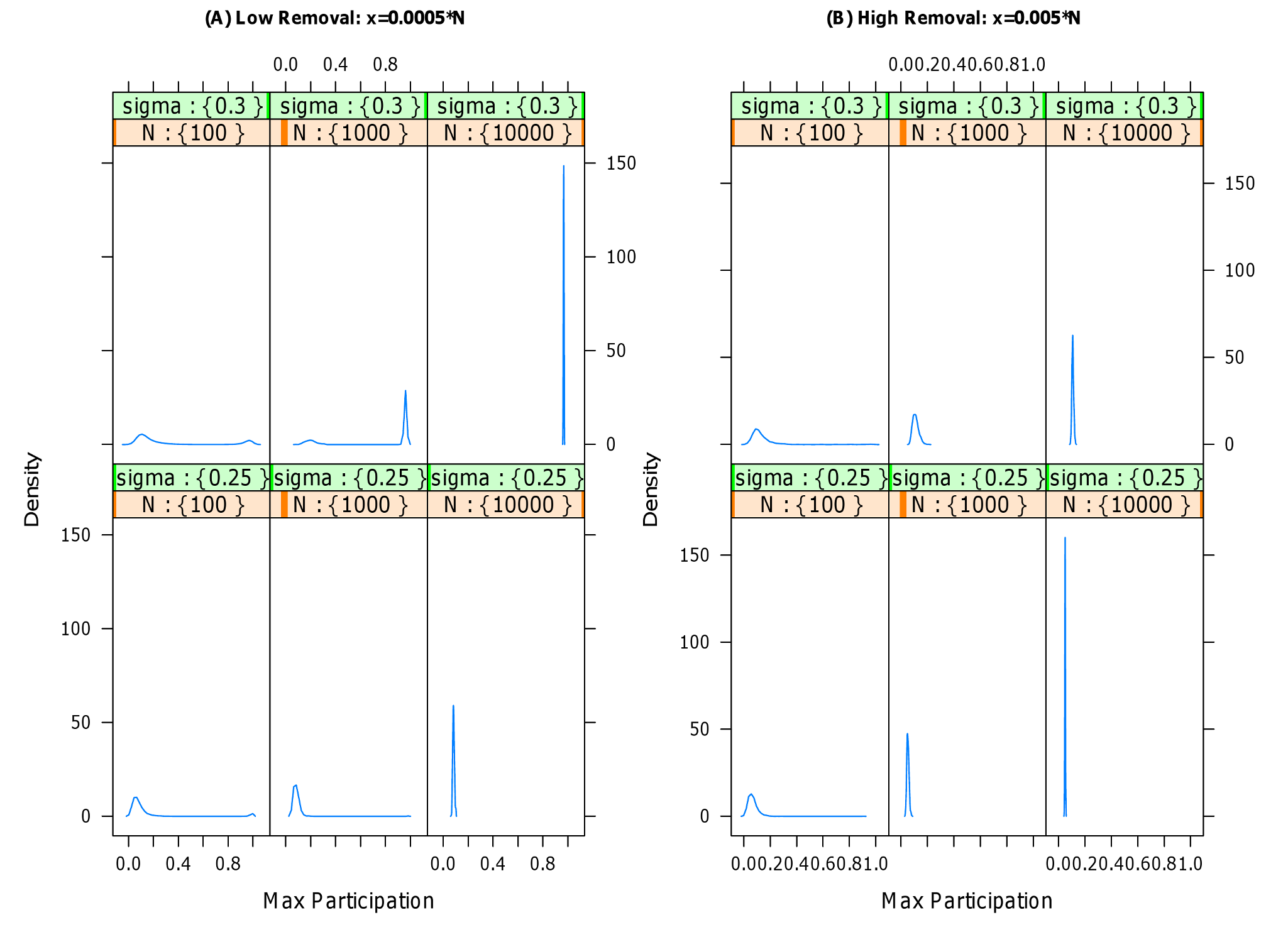

Supplement: Figure S2 — Kernel density plots for the maximal participation level under two rates of removal: (A) Low (x = 0.0005 N) and (B) High (x = 0.005 N). (A) and (B) each contain six subplots that vary N and σ. (TIFF) [file pone.0018545.s002.tif]

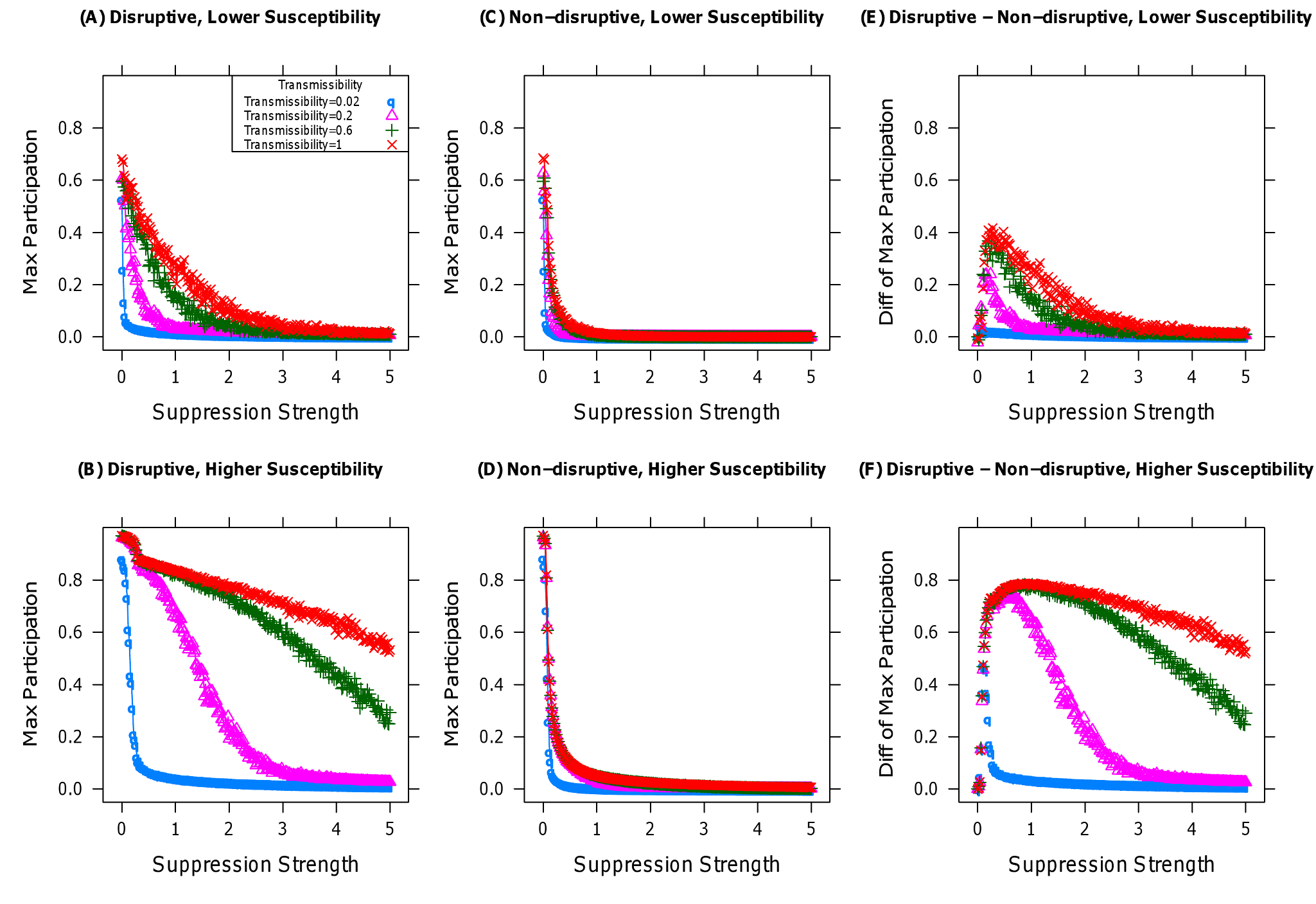

Supplement: Figure S3 — Effect of suppression on mean participation level. (A) and (B) display the effect of disruptive tactics on mean participation levels. (C) and (D) display the effect of non-disruptive tactics on the same. (A) and (C) use populations with lower susceptibilities than (B) and (D); each curve represents a different level of transmissibility. (E) and (F) display the differences between participation under disruptive and non-disruptive tactics (subtracting (C) from (A) and (D) from (B)), using respectively populations with lower and higher susceptibilities. (TIFF) [file pone.0018545.s003.tif]

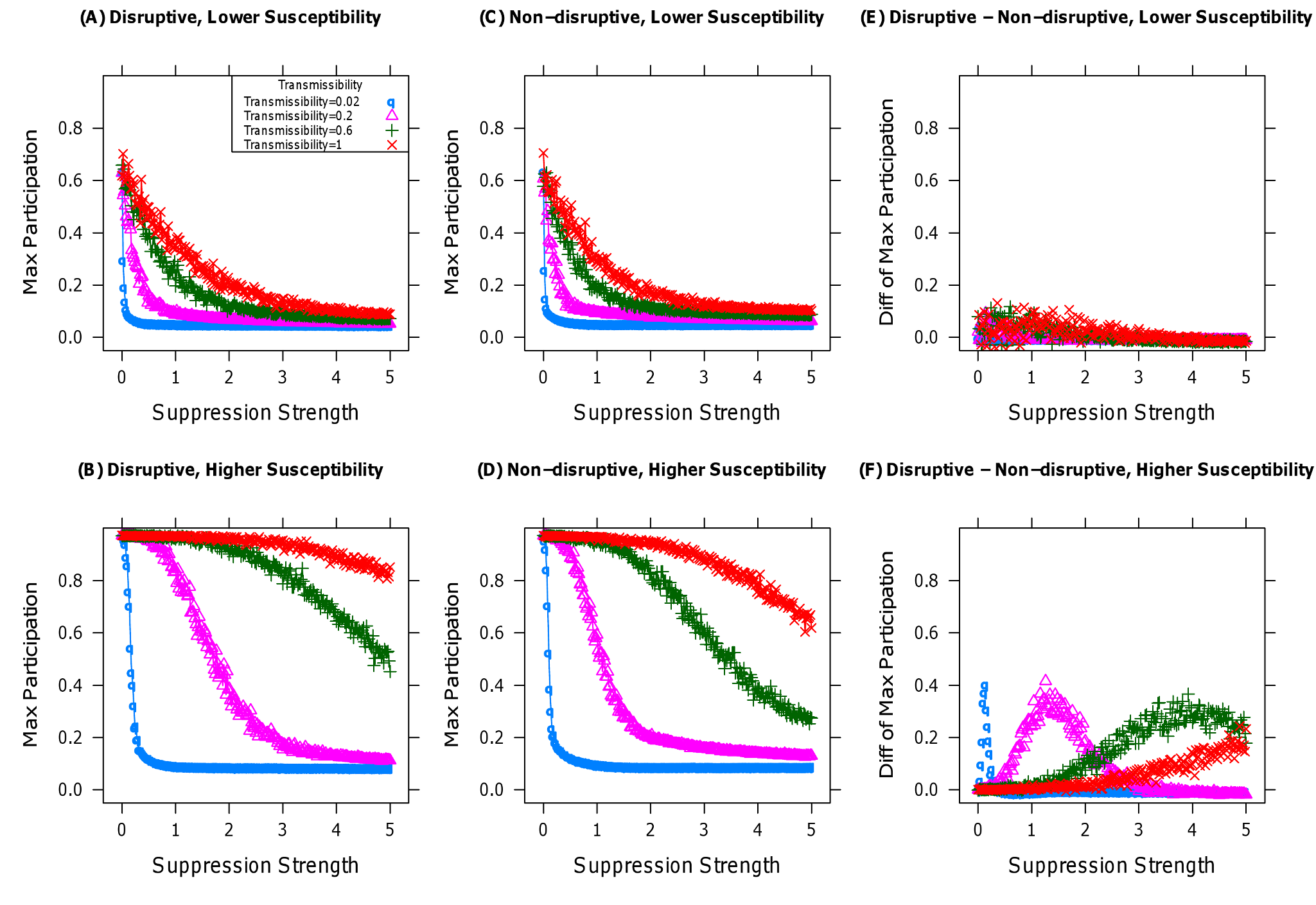

Supplement: Figure S4 — Effect of suppression on maximum participation level; no one can cease participating. (A) and (B) display the effect of disruptive tactics on maximum participation levels. (C) and (D) display the effect of non-disruptive tactics on the same. (A) and (C) use populations with lower susceptibilities than (B) and (D); each curve represents a different level of transmissibility. (E) and (F) display the differences between participation under disruptive and non-disruptive tactics (subtracting (C) from (A) and (D) from (B)), using respectively populations with lower and higher susceptibilities. (TIFF) [file pone.0018545.s004.tif]

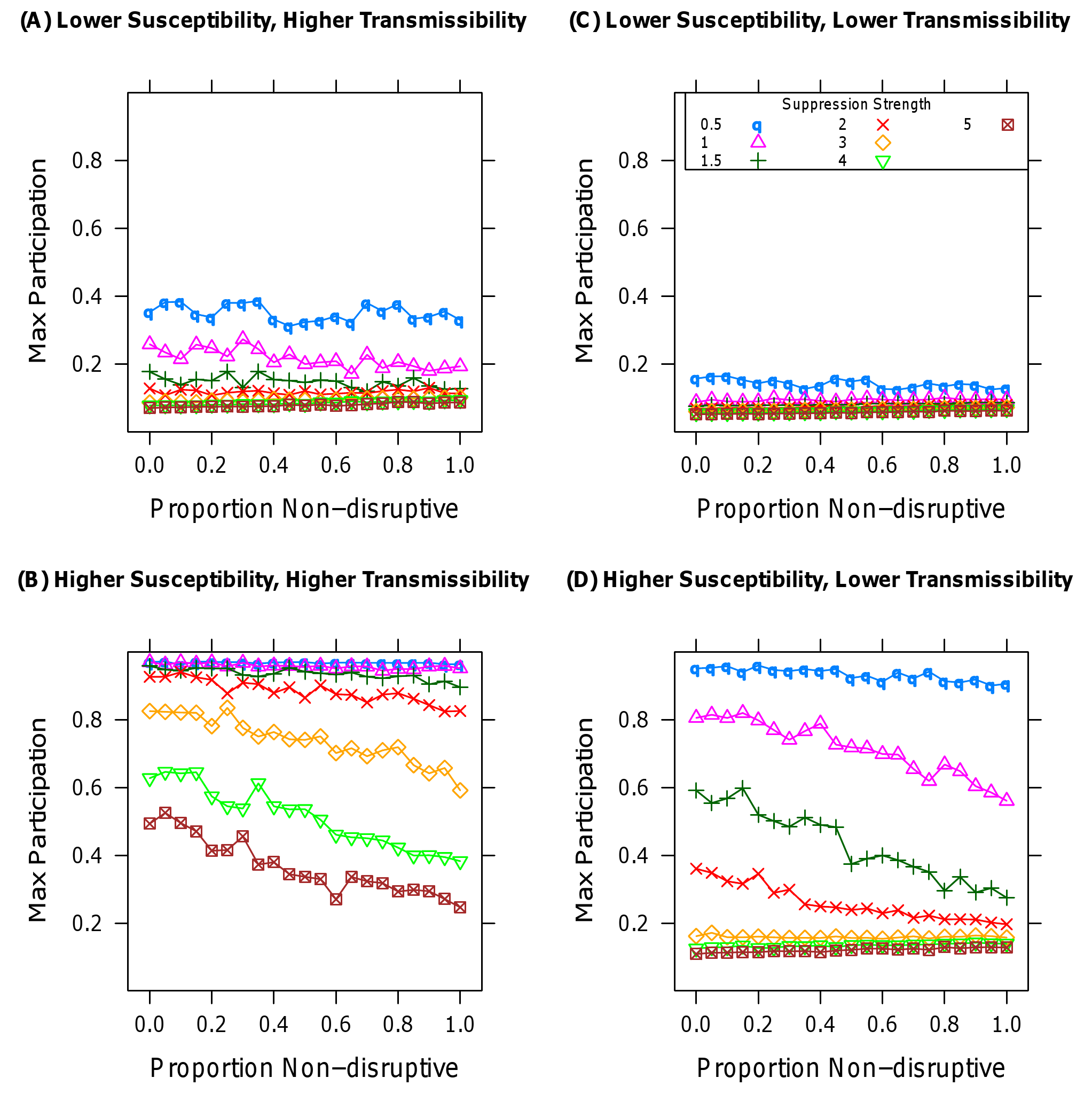

Supplement: Figure S5 — Aggregate maximal participation plotted against the proportion of the total suppression strength applied to non-disruptive tactics; no one can cease participating. Each curve represents a different total suppression strength. (A) and (C) use a population with lower susceptibilities than (B) and (D). (A) and (B) use greater transmissibility than (C) and (D). (TIFF) [file pone.0018545.s005.tif]

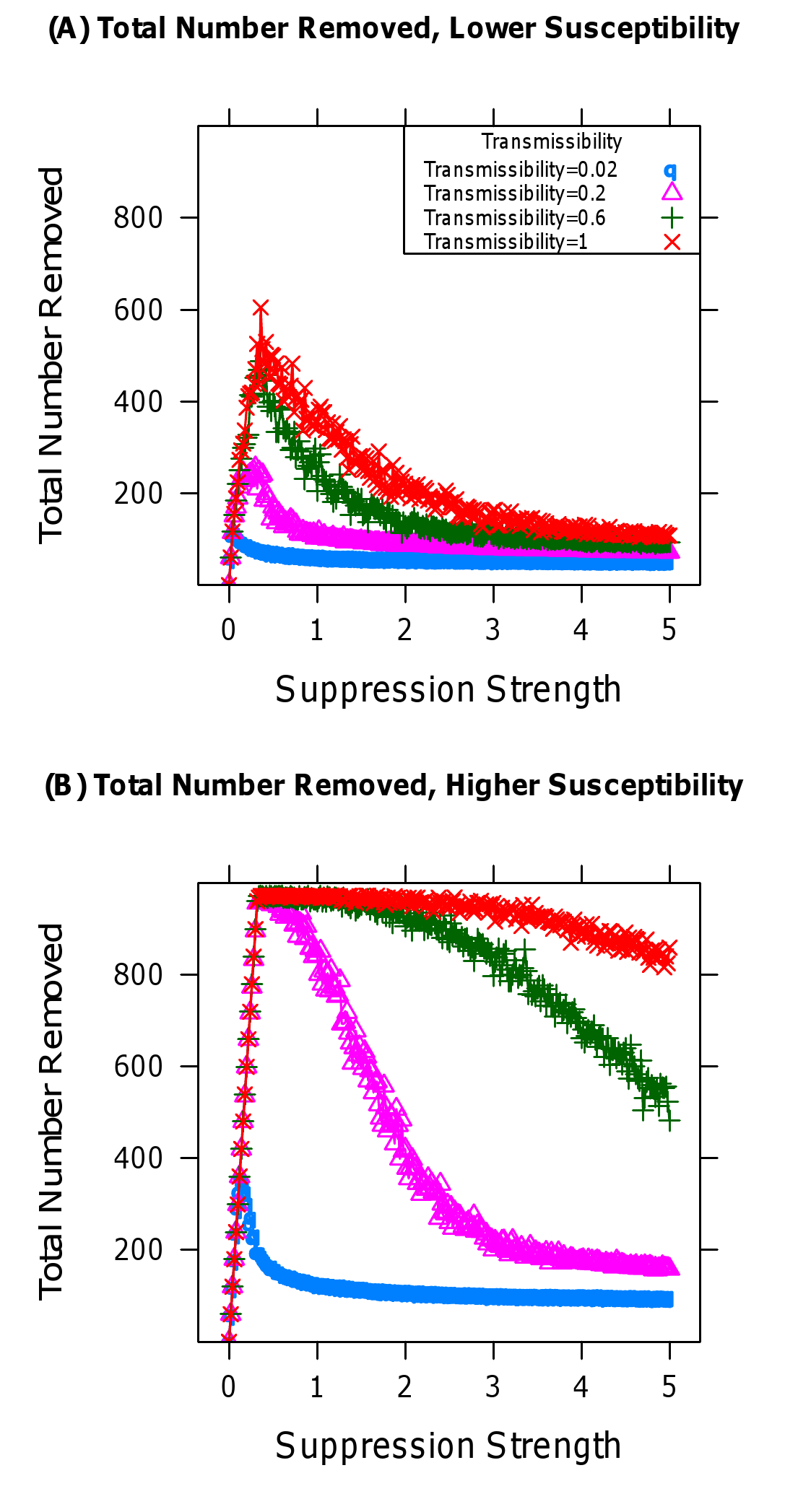

Supplement: Figure S6 — Number of individuals removed; no one can cease participating. (A) and (B) display the number of individuals removed during the period of nonzero participation. (A) uses a population with lower susceptibilities than (B). Each curve assumes a different level of transmissibility. (TIFF) [file pone.0018545.s006.tif]
