# Supplementary material for: Non-Disruptive Tactics of Suppression Are Superior in Countering Terrorism, Insurgency, and Financial Panics
Source: PLoS One. 2011 Apr 13;6(4):e18545. doi: 10.1371/journal.pone.0018545 (PMC3076430; doi:10.1371/journal.pone.0018545)
Supplement: File S1 — Supporting Information (PDF) [file pone.0018545.s007.pdf]

Supporting Information for: *Non-disruptive Tactics of Suppression are Superior in Countering Terrorism, Insurgency, and Financial Panics*

David A. Siegel*

1 Model Description

There are two types of actor in the model: individuals within a finite and heterogeneous population, and an external suppressive entity. I refer to any such entity as a *state* in what follows, as states are the most typical employers of this sort of suppression. Supporting Figure S1 provides a schematic of the order of operations in the model. As one can see, after an initialization stage, in each of a sequence of periods each individual may participate in some collective action. *Participation* equates to supporting or participating in insurgency or terrorism, participating in protests or mass movements, or giving in to a financial panic and divesting oneself of financial instruments. The state desires only to minimize the proportion of the population participating, and can use either disruptive or non-disruptive tactics to do so. State suppression occurs after individual actions within each period. I next describe the assumptions on each type of actor. For simplicity of exposition, I will discuss the model's

*Assistant Professor, Department of Political Science, Florida State University, Tallahassee, FL 32306-2230; e-mail: dsiegel@fsu.edu; phone: 850-645-0083; fax: 850-644-1367; web: `http://myweb.fsu.edu/dsiegel/`.

assumptions first in terms of support for terrorism or insurgency, or participation in protests or mass movements, and then elaborate on the translation of each model parameter to financial panic in a subsequent subsection.

1.1 Population

There are N individuals in the population, each indexed by i. Each person bases her decision to support or participate in terrorism or insurgency, or participate in protests or mass movements, on two mutually exclusive and exhaustive factors. The first factor is the net of personal, idiosyncratic incentives, for or against support or participation, that do not depend on the behavior of others during the series of periods in the model. I call this net internal incentive an individual's *susceptibility* to support or participation, and denote it $b_{i,t}$ for individual i at time t. As the personal incentives that are the constituent components of this net factor are varied and may include for example commitment to the cause, beliefs about state legitimacy, available resources, opportunity costs, and so on, the net of these at the start of the model, $b_{i,0}$, will most likely be the sum of random variables drawn from many different distributions, each corresponding to a different type of personal incentive. Accordingly, I assume that each $b_{i,0}$ is drawn from a normal distribution, with mean μ and standard deviation σ.[1]

The second factor is the net of all incentives that relate to the behavior of others during the series of periods in the model. These might include concerns of fairness if others were to

[1]If there are many personal incentives that are relevant to the decision, draws from the distributions of each of the different types of personal incentives are independent, and the third central moments of these distributions satisfy the Lyapunov condition, then the sum of the random variables drawn from the distributions will be distributed normally, by Lyapunov's central limit theorem. Violations of this limit theorem alter the distribution from which individuals' net incentives are drawn, not the assumption that a single index can represent internal incentives. Different distributions do lead to different aggregate levels of support (see, e.g., Granovetter and Soong 1986; Yin 1998) in the absence of suppression; an investigation of the same under suppression could be a productive avenue of further research.

shoulder a burden that you did not, others' direct influence on your behavior, information gleaned from others' behavior, or an increased feeling of safety the more of the population shares your behavior. I call this net external incentive an individual's *exposure* to the ideas, information, and influence of supporters and participants, and denote it c_t at time t, with $c_t \in [-1, 0]$. Note that all individuals have the same level of exposure in this model. As c_t will be responsive to others' actions, and as $b_{i,t}$ is normally distributed, I do not distribute c_0. Instead I set $c_0 = -1$ for all i. As will be seen shortly, this is the minimum value of c_t, and setting it to this ensures that the model captures the early build up of support without biasing the population toward support. The path dependent nature of the model's dynamics implies suppression of any type is far less effective when it begins after support has already begun to grow. A state unable to apply suppression until after a buildup of support has already happened will experience less effective suppression than exhibited in the paper.

After the initial assignment of $\{b_{i,0}, c_0\}$, individuals take two actions in each period. First, individuals simultaneously update c_t according to the following equation: $c_{t+1} = \lambda c_t - (1 - \lambda)(1 - s_t)$. s_t is the population rate of participation at time t, i.e., the fraction of the total population participating in the collective action.[2] This updating rule specifies an increase in exposure from -1 toward 0 as the rate of participation increases toward 1.[3] It is therefore more applicable to the bandwagon effect associated with growing support than to a model of public goods in which external incentives might begin to decrease at some level of provisioning of the good (c.f., Marwell and Oliver 1993). The form of the rule has three advantages. One, it has few cognitive requirements. Two, it allows for variation in the

[2]An alternative specification, not considered here, would update c_t on the absolute number of other participants. The choice of specification is, in general, a substantive one. Here preference is given to the degree of uniformity of behavior over absolute participation, in line with the common choice in the literature. The degree to which this preference is appropriate will vary by context. A potentially interesting model extension would be to examine the differences in outcome induced by this specification decision.

[3]For example, if $\lambda = 0.5$ and $c_{t-1} = -0.5$, then when $s_t = 1$, $c_t = -0.25$, an increase toward 0, while when $s_t = 0$, $c_t = -0.75$, a decrease toward -1.

speed with which individuals respond to others' actions via the parameter λ. The larger is λ, the lesser one's response to others' behavior; thus it can be thought of as corresponding to one's inertia in decision making. However, to make the argument clearer, in all results I plot instead the value $1 - \lambda$, which represents the transmissibility of ideas, information, and influence between people, and refer to this as the transmissibility parameter. Three, the rule is relatively simple to analyze, and, in the absence of suppression, induces baseline dynamics similar to many threshold models (e.g., Centola and Macy 2007; Granovetter 1978; Granovetter and Soong 1986; Siegel 2009; Yin 1998). Alternate formulations, such as Bayesian updating, lack one or more of these properties. Note that the model displays nonlinear dynamics even with linear components. As it is not the focus of this study, I assume transmissibility is the same for all individuals in the population.

The second action individuals take each period is to make a choice to support or participate according to a simple linear inequality. If $b_{i,t} + c_t > 0$, individual i participates in the collective action at time t; otherwise i does not. Since the c_t are bounded in $[-1, 0]$ by the form of the updating equation above, while the $b_{i,t}$ are not, there can be individuals who will never add their participation, and those who will do so regardless of others' behavior. As there is no a priori reason to choose a nonlinear decision rule, I use the simple rule here to make analysis clearer.

1.2 State

The state, which desires solely to minimize the level of participation in the collective action, has two types of suppression at its disposal: disruptive and non-disruptive. In the context of terrorism and insurgency, these correspond to, respectively, force and hearts and minds. The first, force, involves the removal of an individual from the population. This directly reduces aggregate support by eliminating a participant, and indirectly by reducing others' external incentives. The faster is removal, the stronger the suppression of aggregate support and participation.

I assume that the state can observe only behavior, not individuals' true internal incentives (Kuran 1991). As such, it cannot target specific supporters, and instead removes supporters at random. Given the form of individuals' updating, it is always preferable to remove a supporter to a non-supporter; thus no non-supporters are removed. I assume supporters are identifiable; if they are not removal becomes even less effective. To accommodate different suppressive capabilities, removal happens only at the limited rate $x \geq 0$, corresponding to the maximal number of individuals that may be removed in each period.[4] In all results relating to disruptive suppression tactics, suppression strength refers to the parameter x. As, even given emotional responses, it is always better for the state to remove individuals faster than slower, I assume that the state always removes individuals at its maximal rate. Removing an individual alters s_t, and thus indirectly affects individuals' decisions through their c_t.

The second type of suppression, hearts and minds, is designed to change people's level of support without eliminating individuals. It is therefore equivalent to providing supporters and potential supporters with disincentives to support the collective action, altering their perceived costs and benefits. In the model, the state applies these disincentives by decreasing individuals' internal incentives, $b_{i,t}$, by some fixed amount, $z \geq 0$. The greater is the decrease, the stronger the suppression of aggregate support and participation. In all results relating to non-disruptive suppression tactics, suppression strength refers to the parameter z multiplied by the population size N for scaling purposes. Unlike for removal, it is often not feasible for the state to preclude non-supporters from receiving disincentives, as they often take the form of public goods such as infrastructure development or changes to the legal regime. Further, applying beneficial disincentives only to supporters could perversely induce support from non-supporters eager to receive the state's largesse. Thus disincentives are applied to all

[4]Non-integer values of x are accommodated by accumulating suppressive capability until an integer is reached. A rate of 0.5 thus results in one person removed every two periods, 1.5 three every two periods, and so on.

individuals equally in the model.

Suppression of either type is applied at the end of a period, just after the computation of the aggregate level of participation. Thus, both types of suppression reoccur over time. Should suppression happen all at once—a single crushing attack, the opening of a hospital or, for financial panics, a one-time insurance payment—it would be strictly more effective than the equivalent sum of suppressive acts over time. s_t is adjusted appropriately when removal alters the level of participation. In general, the state continues to suppress until $s_t = 0$ (or everyone has been removed), though see the simulation method section below for additional details.

1.3 Application to Financial Panics

The model applies in a straightforward manner to the spread of financial panic. Support or participation equates to giving in to a panic and withdrawing one's money or divesting oneself of one's financial instruments. s_t represents one's rate of interaction with the panicked; $1 - \lambda$ the transmissibility of the panic; c_t one's effective level of exposure, incorporating transmissibility; and $b_{i,t}$ one's level of susceptibility. The updating rule assumes that panic is more likely the more exposure one has to others who are panicked. The bounds on effective exposure imply that any level of $b_{i,t} \leq 0$ is equivalent to full insurance from financial losses, rendering panic unnecessary. Non-disruptive reductions in $b_{i,t}$ that do not achieve this level correspond to the granting of partial insurance from financial losses, which is the analogue of the disincentives of a hearts-and-minds approach. Preventing a person from exposing others by applying a gag rule to the media, to a financial institution, or directly to a person that prevents anyone from knowing the financial actions of that person, is the analogue of force and a disruptive tactic.

The only significant difference in terms of the application of the model lies in the decision rule. Specifically, the dynamics of recovery from panic may differ from the dynamics of withdrawal of support. As long as the state is not vindictive, one can respond to changing

circumstances by choosing to cease one's support or participation without further incident. These options are often not available in the context of financial panic. While one can return one's money to a bank, assuming it is still solvent, and thus cease "support" for a bank run, it is rarely possible to repurchase a financial instrument at its original value during a panic.

I address this difference by making the simplest specialization of the model to this case: once an individual participates, she is locked in to participate from that point forward. While we could consider more complicated models,[5] this model variant mimics the inability of people to take back their financial transactions, while maintaining the focus on interdependent behavior. Supporting Figures S4, S5, and S6 show that all major results carry through with this variant model.

1.4 Emotional Response Model Assumptions

Another cost to disruptive tactics is the possibility of additional mobilization driven by anger in response to force. To account for this, I introduce a variant of the model in which the removal of any individual creates an emotional response in all others in the population. Each removal induces anger in the rest of the population, which grows with each person removed. Formally, anger shifts susceptibility higher, by an amount equal to a constant, $y_c > 0$, times the total number of people removed to that point. Anger is applied just before the state's removal action, so Floor$[x * (t-1)]$ is the total number of people removed by the beginning of period t. Thus, in period t the susceptibility addition due to anger is $y_t = y_c * \text{Floor}[x * (t-1)]$. As such, the more people are removed, the greater the emotional response. As earlier, though the rule is linear, the response is strongly nonlinear, and so I use the simple linear rule. Though one can imagine cases where a gag rule could lead to anger on the part of the population, this model extension is meant to apply largely to cases

[5]For example, one could allow repurchase at a loss. Formally, this would imply that individuals panic when the sum of their incentives exceed zero, but cease their panic and buy back their financial instruments only when the sum of their incentives drops below some negative cutoff.

of support for or participation in insurgencies or terrorism, or participation in protests or mass movements, where individuals might mobilize in response to state violence.[6]

2 Simulation Method

As I assume that the population consists of a finite number of discrete individuals, rather than modeling it as a continuous distribution, results for the model are obtained via simulation, since there is no closed-form, tractable analytic solution for this case.[7] In this section I discuss both the simulation methodology and the role of population size on the model's outcomes. The section concludes with an extension to the infinite population case, largely to illustrate the difficulties inherent in such an approach.

To derive results from the simulation, I rely on detailed parameter sweeps across regions of the full parameter spaces spanned by $\{N, \mu, \sigma, \lambda, x, (y_c)\}$ for removal (with emotional response) and $\{N, \mu, \sigma, \lambda, z\}$ for disincentives. To choose regions to sweep, I rely on extant theory and supplemental simulations to pare the parameter space of interest. First consider the number of people, N. In similar models, N alters outcomes (e.g., Oliver and Marwell 1988; Siegel 2009), and so we would expect it to alter maximal participation levels here as well. In particular, larger populations should produce more participation when susceptibilities are high, and less when they are low, with the reverse true for smaller populations. This should occur due to the model's strong path-dependent behavior, which produces cascade dynamics. Generally, one of two outcomes occurs: either the population tips over into

[6]One could create a fear effect by letting the sign of y_c be negative. I do not discuss this in the paper, but it generally acts as one would expect, decreasing support in a linear or nonlinear fashion.

[7]While the use of continuous distributions can facilitate the derivation of analytic results (see Jackson and Yariv (2007) for an excellent example of a general model of interdependence in the absence of suppression), it is a modeling choice not without consequence (Durrett and Levin 1994). I feel the more natural assumption given limited real-world populations and limited state capability, particularly when it comes to disruptive suppression, is to assume a finite, discrete population.

near-full participation; or only a low level of participation occurs. This implies a bimodal distribution of outcomes, with the location of the modes dependent on the model's parameters. The mean levels of maximal participation reported in the text are thus effectively proxies for the frequency with which a cascade occurs.[8]

In a small population, minor variations in the realization of the susceptibility distribution or in the choice of whom to remove can induce or destroy cascades, producing variance in outcomes. In a larger population limit behavior begins to obtain, leading to a collapse of the distribution of outcomes. Supporting Figure S2 verifies this logic for this model.

Supporting Figure S2 displays kernel densities for two different levels of removal; the same analysis for non-disruptive tactics yields an identical figure and so is not shown. As $N \to \infty$, for any set of parameter values either a cascade always occurs, or it never does. Decreasing N causes the distribution of maximal participation levels to broaden. Smaller populations create opportunities for a cascade to occur when this would not have been possible in an infinite population, and for one not to occur when this would have been guaranteed under an infinite population. The former raises mean maximal participation levels, while the latter lowers them.

As all other variables either scale with or are unaffected by N, the number of people in the model solely has this limit effect. Thus the role of population in the model is clear, and I fix $N = 1000$ in subsequent parameter sweeps. This value reduces some stochasticity while still allowing analysis of substantively interesting intermediate-susceptibility settings. Note that the loss of these settings under an infinite population due to the aforementioned limit behavior prevents simply inferring finite population behavior from the infinite population case. Nevertheless, at the end of this section I briefly lay out an infinite population version

[8]This bimodal distribution renders standard measures of variance of the mean simulation outcomes, such as standard deviations, less useful. Thus I do not include them in the plots. However, the mean values of all output measures change little when increasing the number of simulation histories taken from 200 to 1000, implying stability in mean values and meaningful results.

of the model, which yields implicit analytic solutions from which numerical comparative statics could in principle be derived, though doing so would add little insight.

Next we turn to the internal susceptibility distribution parameters, $\{\mu, \sigma\}$. These act together in determining the native willingness of the population to participate in the collective action. However, varying μ is of little interest as it has the clear effect of uniformly increasing participation in all cases, under all other parameter values. Thus I fix it at $\mu = 0.6$, which allows the variance of other parameters to cover the full range of behavior in the model. The standard deviation, σ, is of more interest, as it has a nonlinear effect. Below a cutoff, increasing it increases participation, while above a cutoff increasing it decreases participation. This cutoff depends on the mean of the distribution: the lower is μ, the greater the cutoff in σ.

When σ is below the cutoff—the more interesting case, as values above the cutoff lead to larger groups of people unaffected in any way by their peers' behavior—there are three regions of interest in σ. In the absence of suppression, low values of σ yield populations that rarely achieve cascades, high values yield populations that usually achieve cascades, and intermediate values yield populations that sometimes yield cascades. Since failure to achieve significant participation absent suppression implies little role for suppression, I do not consider low values of σ in this paper. I choose values of 0.25 and 0.3 to represent lower and higher susceptibilities in the paper; these correspond to regions of occasional and usual cascades, respectively. Other values of σ within these regions yield similar dependences of participation on the other parameters.

I explicitly sweep the other parameters λ, x, y_c, and z. λ has its most interesting effect for very high values; thus I display more of the higher values of λ (lower values of $1 - \lambda$) in the text's figures. All results from these sweeps were obtained by averaging across 200 simulations runs for each set of parameter values. Each simulation run continues until the aggregate level of participation is zero, everyone has been removed, or 3000 periods have passed and neither of the previous two conditions has yet occurred. The last condition is

invoked only for very small disincentive sizes or removal rates, and limits computational time when there is a long, slow decline from maximal participation. It does not change the level of maximal participation.

2.1 Infinite Population Model

This subsection briefly describes an infinite population version of the model of this paper, in which the population may be treated as a continuous distribution over susceptibilities. It is included solely to illustrate how such an analytic model might be set up and, particularly in the case of removal, the difficulty of deriving comparative statics of equivalent utility to those in the text. For simplicity I assume perfect transmissibility.

Begin by considering participation in the absence of suppression. In this case, participation monotonically increases toward its steady state, which occurs at the maximum level of participation obtainable in the population. This occurs when c_t is at its steady state of $\tilde{c} = -(1 - \tilde{s})$ and exactly $\tilde{s}$ fraction of the population has $b_{i,t} \geq -\tilde{c}$, so that:

$$\tilde{s} = 1 - \Phi\left(\frac{1 - \tilde{s} - \mu}{\sigma}\right).$$

Now consider the application of disincentives. Disincentives decrease susceptibility by z in every period. Moving to an infinite population does not change this. This implies that the transition rule between periods in the model under non-disruptive tactics is:

$$s_t = 1 - \Phi\left(\frac{1 - s_{t-1} - \mu + zt}{\sigma}\right).$$

Note that s_t is strictly decreasing in z. The only steady state is at $\tilde{s} = 0$, as disincentives inevitably drive the system toward no participation. The maximum level of participation occurs the first time $s_t < s_{t-1}$ due to the monotonicity of the process. Define $\epsilon_t = s_{t-1} - s_t$. The first $\epsilon_t > 0$ specifies the maximum at $t - 1$. One can solve for this numerically by iterating the definition of ϵ_t.

Finally, consider removal. Assume that, in a population defined by a continuous distribution of "types", i.e., levels of susceptibilities, removal equates to the removal of a convex

set of types of strictly positive measure from the distribution of susceptibilities. Define θ_t as the minimum type removed in period t, and ϕ as the measure of the set of types removed. Since removal is random, θ_t is a random variable. Let its cdf be Θ_t, with $\Theta_t = 0 \; \forall \; \theta_t \leq 1 - s_t$, and $\forall \; \theta_t \in [\theta_j, \theta_j + \phi]$ with $j < t$. Then we have that:

$$s_t = \int_{\Omega_{t-1}} d\Phi(s_{t-1}; \mu, \sigma),$$

where

$$\Omega_{t-1} = (1 - s_{t-1}, \infty) \backslash ([\theta_1, \theta_1 + \phi] \times \ldots \times [\theta_{t-1}, \theta_{t-1} + \phi]).$$

Note that s_t is decreasing in ϕ, giving us this comparative static, but that an analytic comparison between removal and disincentives in this framework is untenable. This serves as another reason to turn to simulation, given the ability of the simulation model to make explicit comparisons between suppression tactics.

3 Effect of Suppression on Mean Level of Support

The maximal participation level discussed in the text has direct relevance for state stability under insurgency, terrorism, protest, and mass movements, and state policy in runs on financial institutions. When the state is at risk of failing, measures such as the mean that include aggregate behavior after the maximal participation level has been reached place too much weight on behavior potentially occurring after the fall of the state. Assuming the fall of the state changes the nature and target of participation, the mean is thus not an appropriate measure. Nor is the mean a particularly good measure for many financial panics, given the comparative irreversibility of individual behavior in these cases. However, when the collective action has no chance of toppling the state, the mean level has meaning and its analysis yields additional insight.

Supporting Figure S3 replicates Figure 1 in the text using the mean participation level rather than the maximum. The results for removal are qualitatively the same between the two

figures, but disincentives are far more effective in minimizing the mean level of participation than they were in minimizing the maximum level of participation. The reason for this is that co-opting former supporters contributes to suppression even after the maximum level of participation has been reached. The same is not true under removal. Removing individuals from a fully-mobilized population does little to change the context of behavior going forward—everyone still sees nearly the entire population supporting the collective action. Thus, when the state is unlikely to fall, hearts-and-minds approaches are even more preferable to force.

As noted, in contrast to the text's Figure 1, this result does not apply directly to many financial panics, as the model does not include true mechanisms for individuals to reacquire the financial instruments they abandoned. However, some conclusions can be drawn. When there is no individual left in the population who is susceptible to panic, gag rules are worthless. Consequently, any level of insurance that carries with it the potential for recovery of financial wellbeing is trivially superior. Of course, this outcome is not due to interdependency. It is also worth noting that the result in Supporting Figure S3 does apply directly to any case in which financial recovery is possible, like a bank run in which the bank remains solvent.

4 References

Centola, Damon and Michael Macy. 2007. "Complex Contagions and the Weakness of Long Ties." *American Journal of Sociology* 113(3): 702–734.

Durrett, Richard and Simon Levin. 1994. "The Importance of Being Discrete (and Spatial)." *Theoretical Population Biology* 46: 363–394.

Granovetter, Mark S. 1978. "Threshold Models of Collective Behavior." *American Journal of Sociology* 83(6): 1420–1443.

Granovetter, Mark S. and Roland Soong. 1986. "Threshold Models of Interpersonal Effects in Consumer Demand." *Journal of Economic Behavior & Organization* 7(1): 83–99.

Jackson, Matthew O. and Leeat Yariv. 2007. "Diffusion of Behavior and Equilibrium Properties in Network Games." *American Economic Review (Papers and Proceedings)* 97(2): 92–98.

Marwell, Gerald and Pamela E. Oliver. 1993. *The Critical Mass in Collective Action: A Microsocial Theory*. Studies in Rationality and Social Change. Cambridge: Cambridge University Press.

Oliver, Pamela E. and Gerald Marwell. 1988. "The Paradox of Group-Size in Collective Action–A Theory of the Critical Mass 2." *American Sociological Review* 53(1): 1–8.

Siegel, David A. 2009. "Social Networks and Collective Action." *American Journal of Political Science* 53(1): 122-138.

Yin, Chien-Chung. 1998. "Equilibria of Collective Action in Different Distributions of Protest Thresholds." *Public Choice* 97(4): 535-567.
